# A highly conspicuous mineralized composite photonic architecture in the translucent shell of the blue-rayed limpet

**DOI:** 10.1038/ncomms7322

**Published:** 2015-02-26

**Authors:** Ling Li, Stefan Kolle, James C. Weaver, Christine Ortiz, Joanna Aizenberg, Mathias Kolle

**Affiliations:** 1 Department of Materials Science and Engineering, Massachusetts Institute of Technology, 77 Massachusetts Avenue, Cambridge, Massachusetts 02139, USA; 2 Wyss Institute for Biologically Inspired Engineering, Harvard University, 60 Oxford Street, Cambridge, Massachusetts 02138, USA; 3 School of Engineering and Applied Sciences, Harvard University, 9 Oxford Street, Cambridge, Massachusetts 02138, USA; 4 Kavli Institute for Bionano Science and Technology at Harvard University, 29 Oxford Street, Cambridge, Massachusetts 02138, USA; 5 Department of Mechanical Engineering, Massachusetts Institute of Technology, 77 Massachusetts Avenue, Cambridge, Massachusetts 02139, USA

## Abstract

Many species rely on diverse selections of entirely organic photonic structures for the manipulation of light and the display of striking colours. Here we report the discovery of a mineralized hierarchical photonic architecture embedded within the translucent shell of the blue-rayed limpet *Patella pellucida*. The bright colour of the limpet’s stripes originates from light interference in a periodically layered zig-zag architecture of crystallographically co-oriented calcite lamellae. Beneath the photonic multilayer, a disordered array of light-absorbing particles provides contrast for the blue colour. This unique mineralized manifestation of a synergy of two distinct optical elements at specific locations within the continuum of the limpet’s translucent protective shell ensures the vivid shine of the blue stripes, which can be perceived under water from a wide range of viewing angles. The stripes’ reflection band coincides with the spectral range of minimal light absorption in sea water, raising intriguing questions regarding their functional significance.

## Abstract (web summary)

Mollusks have evolved an exquisite diversity of complex mineralized shells for protection. One such example, the blue-rayed limpet, incorporates a vivid display of blue lines, which originate from the interference of light in a nano-periodic photonic architecture buried within the animal's translucent shell.

## Graphical abstract

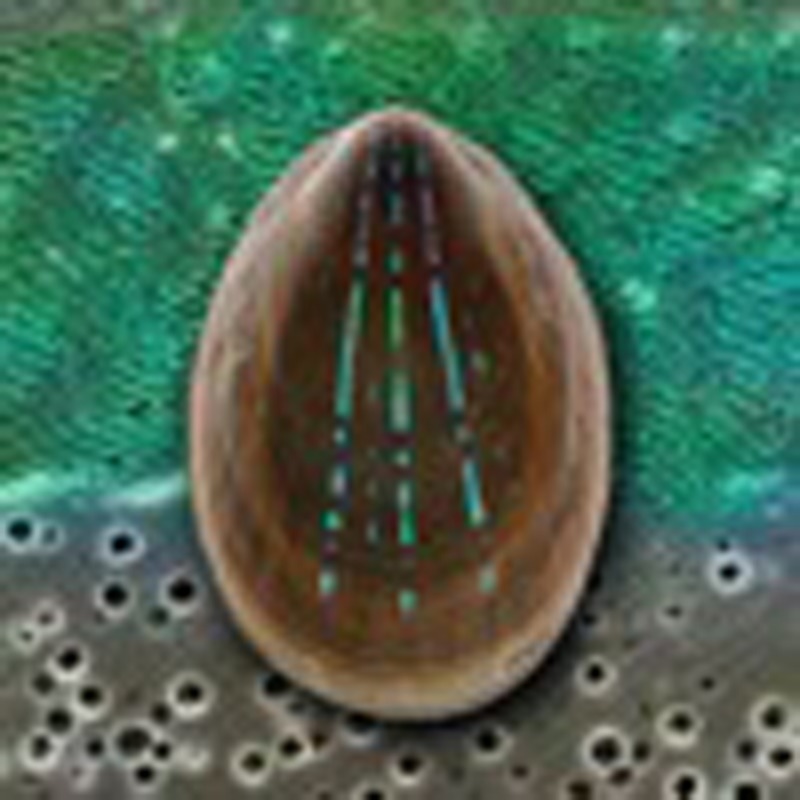

Through the course of evolution, many species have developed ingenious ways to use light to create unique visual displays^1^,^2^,^3^,^4^,^5^. Pigment-based spectrally selective absorption empowers air-borne, terrestrial and aquatic creatures to display mostly red, orange, yellow and more rarely green or blue hues in their skins, plumages, scales or shells^6^,^7^,^8^. In contrast, nano- or micro-periodic organic structural architectures have evolved to interfere with light where strong metallic reflections and iridescent colorations are required or the pigmentation for a specific hue is unavailable^6^,^9^. Most natural occurrences of blue colour, for example, are due to the interaction of light with such biological photonic systems. Structural blues have been found in the feathers of peacocks and other bird species^10^,^11^,^12^,^13^, the wing scales of butterflies and moths^2^,^4^, the exoskeletons of beetles^14^,^15^, in the skins of birds and mammals^16^,^17^ and even in the skin of fruits^18^,^19^, shedding light on a stunning diversity of biologically evolved light manipulation mechanisms.

Despite the fact that structural colour is also extremely common in marine ecosystems, with representatives including algal, invertebrate and vertebrate species, few examples have been well characterized, and in-depth investigations have been limited to metallic fish scales^20^,^21^,^22^, the reflecting setae of crustaceans^23^ and polychaetes^24^,^25^ and, most notably, the camouflage and coloration control mechanisms of cephalopods that rely on the intriguing interplay of localized dynamic light-absorbing chromatophores, iridescent iridophores and strongly scattering leucophores^26^,^27^,^28^.

The majority of structurally diverse, functional biophotonic architectures in different species are mainly comprised of highly ordered organic materials, including chitin, guanine, collagen, keratin, reflectin, pterin, melanin or carotenoids^21^,^22^,^29^,^30^,^31^,^32^,^33^,^34^. Being the most prominent example of a biologically produced mineral-based iridescent material, nacre’s diverse colour palette originates from light interference within its layered composite structure of microscopic aragonite tablets. The structural colour is only apparent in the interior of the shell with little to no external visibility. Most likely mechanical robustness^35^ is the primary biological purpose of this laminated microstructure.

Here we describe a localized, structurally complex and entirely mineralized photonic system embedded within the continuum of a translucent mollusk shell that lies at the origin of the striking visual appearance of the blue-rayed limpet, *Patella pellucida*. The blue coloration of the limpet’s stripes is a structural colour that is the result of the interference of incident light in a composite photonic architecture, which is buried within the shell. This photonic structure consists of a nanoscale-periodic layered arrangement of crystallographically co-oriented calcite lamellae with regular thickness and spacing, which selectively reflects in the blue and green spectral range. Underneath the calcite multilayer, a disordered assembly of light-absorbing particles attenuates light that is transmitted by the multilayer, thereby ensuring saturation of the reflected colour and contrast against the limpet’s white body underneath its translucent shell. The existence of the mineralized photonic architecture in the limpet’s shell proves that a species is capable of exploiting photonic design principles commonly realized by other organisms, which use predominantly organic materials, while concurrently harnessing the superior mechanical properties of inorganic materials to form an armour, which not only provides mechanical protection but also incorporates visual display features. The mineralized photonic architecture likely has evolved to satisfy an optical purpose without overly compromising the shell’s mechanical performance. The underlying design principles could inspire and inform the technological generation of transparent, mechanically robust, multifunctional optical surfaces with incorporated, controllable display capacity.

## Results

### Initial observations

**Figure 1:**
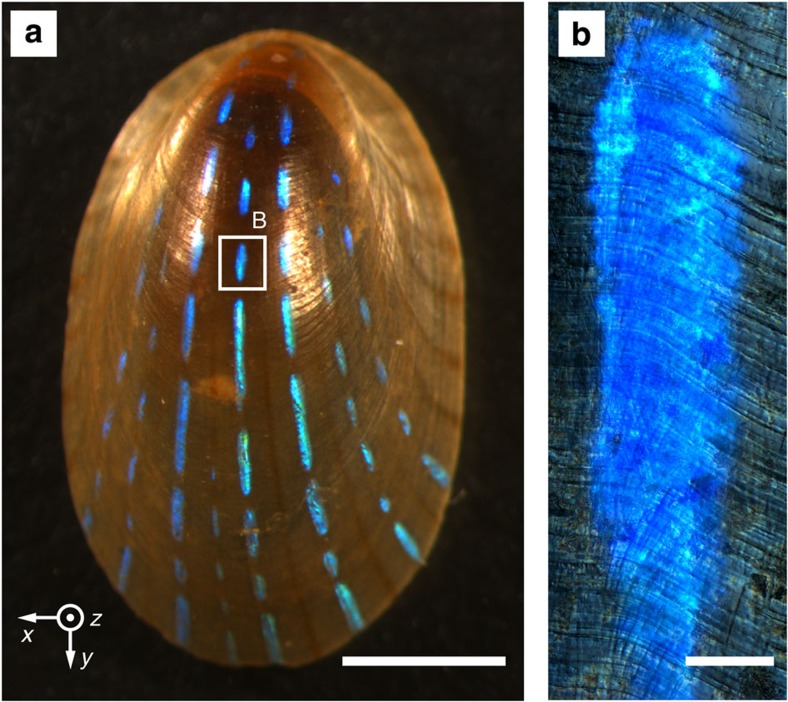
The blue-rayed limpet *Patella pellucida*. (**a**) Optical image of a limpet shell showing the reflection of light from the shell exterior. Scale bar, 2 mm. (**b**) Reflection optical micrograph of a single stripe. Scale bar, 100 μm.

Individuals of the species *P. pellucida* display a dramatic array of thin bright blue stripes along the length of its translucent shell (Fig. 1a). Ranging from coastal Norway and Iceland south to Portugal and west to the Canary Islands^36^,^37^, this species occupies the lower intertidal and subtidal zones of rocky shores (depth <27 m), where it populates the fronds and stipes of *Laminaria*^36^ and other species of large macroalgae. The limpets, occurring both solitarily and in groups, feed on the kelp leaving distinctive circular feeding marks^38^ (Supplementary Fig. 1). The blue stripes first appear as a spotty, interrupted pattern in juvenile limpets (shell length ~2 mm) and become more continuous as the animal grows, although the width of the stripes remains relatively constant (0.1–0.2 mm, Fig. 1b) throughout the life of the animal. The stripe patterns appear to be unique from limpet to limpet and the stripe colour varies from deep blue to turquoise among different individuals. Scanning electron microscopy (SEM) analysis of the exterior and interior surfaces of the shell reveal no distinctive morphological features coinciding with the blue stripes, suggesting that the source of the colour is subsurface (Supplementary Fig. 2). Immersion of a partially damaged stripe in water or index-matching oil results in a shift of the colour towards higher wavelengths or a complete disappearance of the reflected hue, respectively (Supplementary Fig. 3), indicating a colour of structural origin.

### Structural analysis

**Figure 2:**
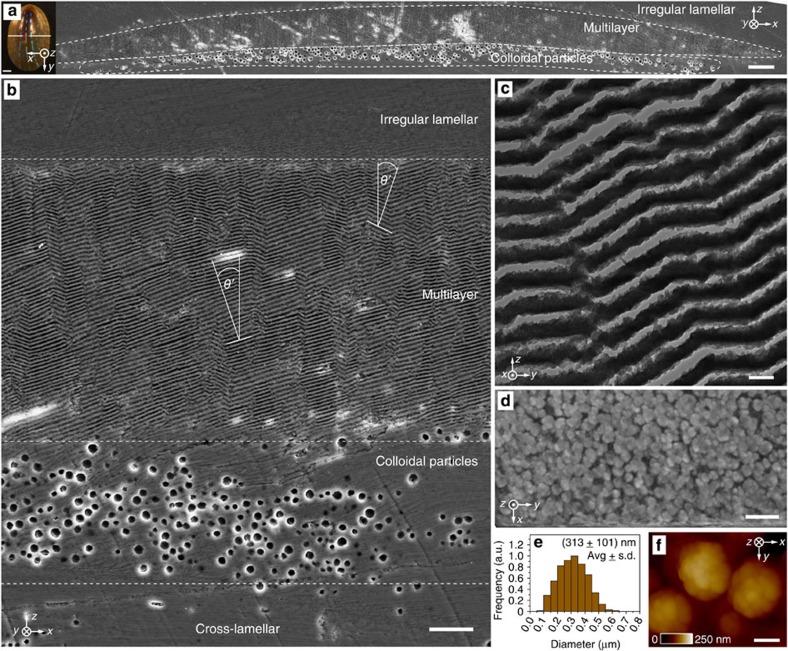
The photonic architecture in the shell of the blue-rayed limpet. (**a**,**b**) SEM images after sectioning and polishing along the white line of the limpet shell denoted in the upper left inset (the centre part of the polishing line, marked in red, represents the area of the cross-section shown in (**a**)). Scale bars, (**a**) 5 μm, inset 1 mm, (**b**) 2 μm. The two different structural components—a regular multilayer on top of a disordered array of colloidal particles (marked with the white dashed lines)—are embedded within the normal densely packed lamellar microstructure, which lacks the detectable gap spacing present in the multilayer region. The characteristic angle *θ*’ between the local multilayer surface normal and the shell surface normal is marked in two positions (**b**). For complete description of the shell’s microstructural regions, please refer to Supplementary Fig. 4. (**c**) TEM image showing the regularity in width and spacing of the mineralized lamellae in the multilayer region. Scale bar, 200 nm. (**d**) Plan-view z-stack overlay showing the distribution of colloidal particles underneath the multilayer, based on X-ray nanotomography reconstructions. Scale bar, 2 μm. (**e**) Size distribution of the colloidal particles. (**f**) Atomic force microscopy height image of the intact colloidal particles, demonstrating their non-faceted fused-granular surface morphology. Scale bar, 100 nm.

In the zones where blue stripes are present, a distinct multilayered structure (Fig. 2a,b) with regular gap spacing between individual lamellae (Fig. 2c) is located at a depth of 10–20 μm beneath the outer irregular lamellar shell layer^39^, where the mineralized building blocks are closely packed without any detectable spacing. The maximum thickness of this multilayer region is ~7–10 μm at the centre (corresponding to 40–60 layers) and gradually decreases in thickness towards the edge of each stripe (Fig. 2a). Beneath this multilayer region, a disordered array (~5 μm in thickness) of colloidal particles is observed (Fig. 2a,b,d–f). Its lateral width (*x* direction in the reference coordinate system) is found to be just short of matching that of the multilayer, leaving the stripe edges free of particles underneath. This distinctive combination of structural features specifically coincides with the location of the blue stripes and is clearly visible in SEM images of cryo-fractured shells prepared from freshly collected specimens, excluding the possibility that these features represent polishing-induced artifacts (Supplementary Fig. 5). The width of the multilayer region (~100 μm) is consistent with the width of the blue stripes observed from optical microscopy studies, further indicating that this is the structure responsible for the optical effects.

Transmission electron microscopy (TEM) imaging of thin sections from the multilayer structure prepared using focused ion beam milling (FIB, Supplementary Fig. 6) reveals that the individual lamellae in the multilayer microstructure have a thickness of 113±11 nm (average±s.d., *n*=52) with the inter-lamella gap spacing measuring 53±7 nm (*n*=29; Fig. 2c, Supplementary Fig. 7). Under physiological conditions, the space between the lamellae is likely occupied with water only or a low-density heavily hydrated organic material. The constant thickness and spacing of the lamellae are maintained throughout the entire multilayer region. In particular, the thickness of the multilayer region decreases towards its edges by reducing the number of lamellae rather than the thickness of individual lamella or the spacing between them.

In general, the multilayer lamellae do not adopt a single global orientation parallel to the shell surface. Instead, the multilayer structure exhibits a complex zig-zag-like architecture with the multilayer surface normal spanning an angle *θ*′ of about 16°–18° with respect to the overall shell surface normal (Figs 2b, Supplementary Fig. 8, and explanations below). This is in contrast to most other photonic multilayer systems found in nature, including the layered morphologies observed in jewel and scarab beetles^40^,^41^,^42^,^43^. When viewed in cross-section, the characteristic angle between the lamellae forming the zig-zag pattern is 144.4°±3.0° (*n*=52). Edge-dislocation-like interconnecting junctions and nanoscopic bridges linking adjacent lamellae observed within the complex zig-zag structure appear to reinforce the mechanical stability of this porous layered architecture (Supplementary Fig. 9)^44^,^45^.

Another unique feature of the blue-rayed limpet’s photonic system is the presence of the disordered array of colloidal particles immediately beneath the multilayer structure (Fig. 2b,d) with an average particle diameter of 300±100 nm (*n*=349, Fig. 2e). Using X-ray nanotomographic reconstructions (see Methods), the colloidal particle array was segmented in the *z* direction and the location of each colloidal particle was spatially mapped relative to its neighbour. Using this technique, a flattened *z* stack that showed the plan-view distribution of the colloidal particles suggested almost complete areal coverage (Fig. 2d). Atomic force microscopy performed on intact colloidal particles in fractured shell cross-sections revealed a complex surface morphology, resembling the agglomeration of smaller granules (~50 nm in diameter, Fig. 2f).

**Figure 3:**
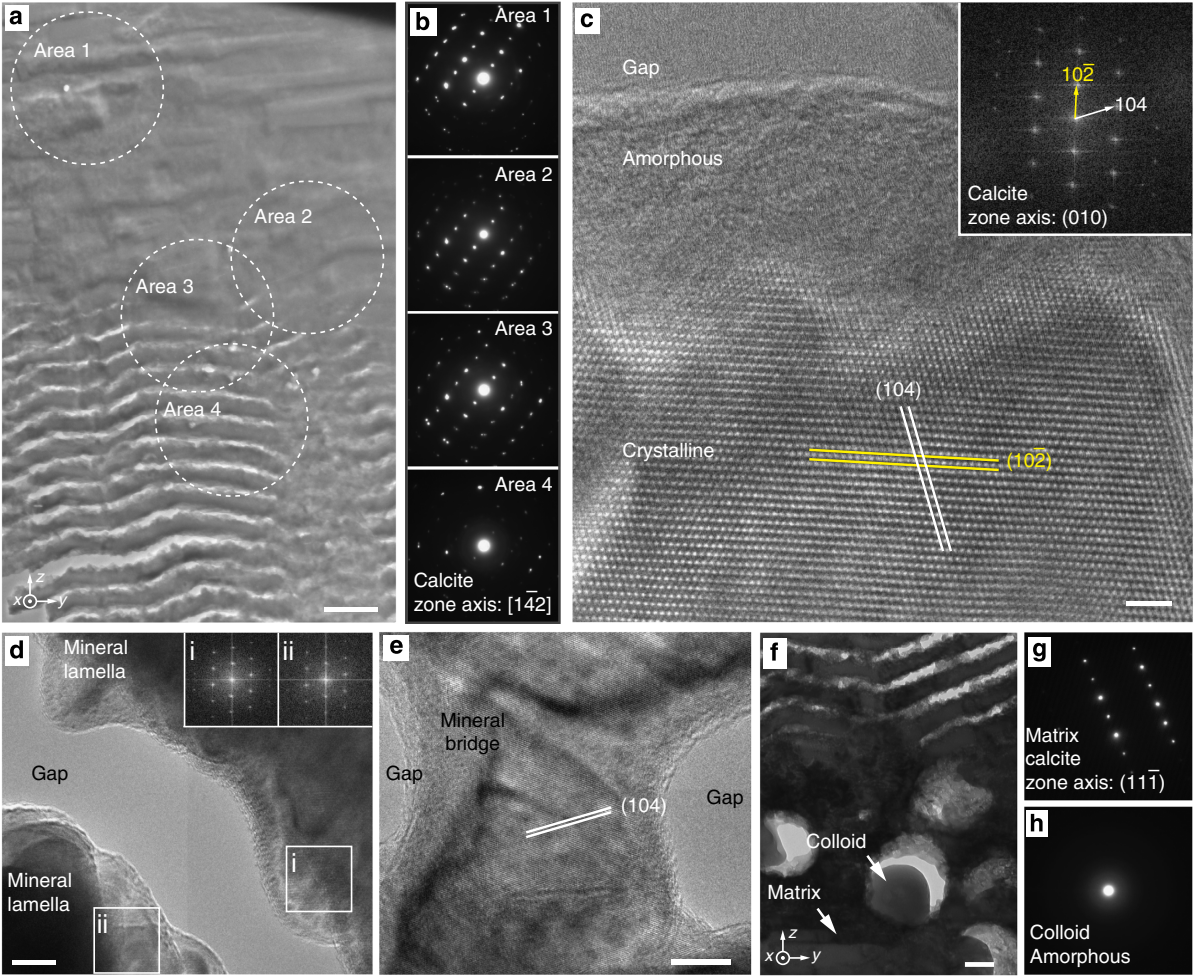
Crystallographic composition of the photonic architecture. (**a**) TEM image of the upper transition zone between the dense lamellar matrix and the photonic multilayer. Scale bar, 400 nm. (**b**) Selected-area electron diffraction (SAED) patterns acquired from the areas marked in **a**, which are all indexed to calcite with a zone axis of 
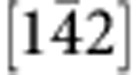
. This demonstrates that both the crystallographic phase and orientation are conserved across the transition zone. (**c**) High-resolution TEM image of the calcite crystal lattice in a single lamella. The top ~10 nm of the lamella appears to be amorphous while the remainder is crystalline. The inset shows the corresponding fast Fourier transform (FFT) pattern of the crystalline region (calcite with zone axis, [010]). Scale bar, 2 nm. (**d**) Parts of two lamellae from the photonic multilayer that are separated by a gap. FFT patterns of the adjacent edges (i, ii) are displayed in the inset, showing that the crystal orientation is preserved across the gap. Scale bar, 10 nm. (**e**) High-resolution TEM image showing a mineral bridge connecting two lamellae within the photonic multilayer, with the crystal lattice translating from one lamella to the next via the connection. Scale bar, 10 nm. (**f**) TEM image of the transition zone between the multilayer and the colloidal particle array. Scale bar, 200 nm. (**g**,**h**) SAED patterns of the surrounding crystalline matrix and amorphous colloids obtained from areas indicated in **f**.

In most mollusk shells, crystalline calcium carbonate (either in the form of calcite, aragonite or a combination thereof) is the primary building material^45^,^46^. As is the case for other limpet species^39^,^47^, the blue-rayed limpet also demonstrates the co-presence of both calcite and aragonite in its shell, as confirmed by whole-shell powder X-ray diffraction (Supplementary Fig. 10). Local crystallographic characteristics in the multilayer and surrounding regions were studied using selected-area electron diffraction and high-resolution TEM imaging (Fig. 3). Diffraction patterns acquired from multiple adjacent multilayer lamellae revealed well-defined single crystal-like diffraction patterns consistent with the calcite polymorph. Multiple closely matched electron diffraction patterns acquired in the transition zone between the multilayer and the top irregular lamellar microstructure indicate that the photonic multilayer not only preserves the crystallographic phase but also preserves the orientation from the surrounding non-photonic regions, despite the presence of 50 nm wide gaps in the multilayer and 300-nm sized particle beneath it (Fig. 3a,b). This conservation of crystal orientation across multiple types of microstructures is known from other mollusk shells^48^; however, most of these microstructures are densely packed with the respective building blocks without such gap spacing or spherical particles being incorporated into the crystals.

Lattice imaging of the calcitic lamellae using high-resolution TEM further illustrates their crystalline nature (Fig. 3c), and corresponding fast Fourier transforms (FFTs, Fig. 3c, inset) were indexed to the (010) zone axis of calcite. In some regions, a thin amorphous layer with thickness of ~10 nm was observed on the surface of the crystalline lamellae (Fig. 3c), whereas in other areas, the lamellae are entirely crystalline (Supplementary Fig. 11). High-resolution TEM images and corresponding FFT patterns obtained from two neighbouring lamellae further confirm the local crystallographic co-alignment in the multilayer region (Fig. 3d). This crystallographic continuity across lamellae is also manifested by the continuous lattice fringes running across the mineral bridges between the adjacent lamellae (Fig. 3e). The crystalline mineral phase continues to be conserved in the matrix surrounding the disordered array of particles beneath the multilayer structure (Fig. 3f,g). In contrast, electron diffraction measurements on single colloidal particles reveal a characteristic amorphous diffraction halo, confirming their non-crystalline nature (Fig. 3f,h).

### Optical analysis

**Figure 4:**
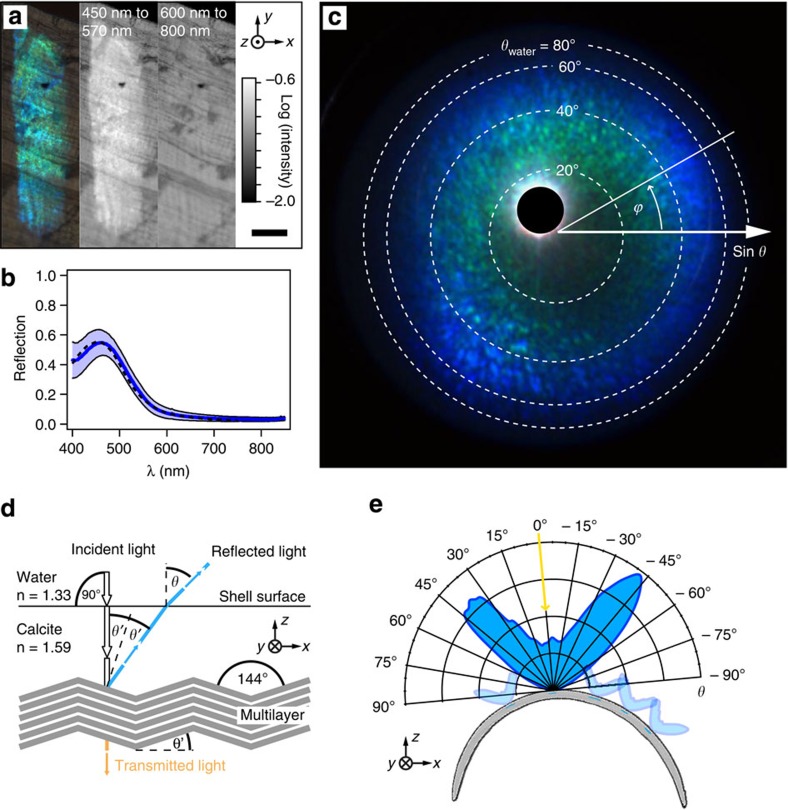
Optical properties of the photonic multilayer. (**a**) Reflection optical micrograph of a single stripe (left) in comparison with the two intensity maps of light reflected in the range of 450–570 nm (middle) and 600–800 nm (right) wavelengths. Scale bar, 50 μm. (**b**) Reflection spectrum of a stripe in water, referenced against a 97% reflective silver mirror. The blue line represents the average reflectivity with the s.d. being visualized by the blue shaded area (seven limpets, 10–20 measurements per shell). The black dashed curve represents theoretical calculations of the blue stripes’ reflectivity that results from 500 calculation runs assuming a multilayer stack with 40 calcite lamellae (*n*=1.59) and water filling the interstitial gaps (*n*=1.33) with a lamellae thickness of 113±11 nm and a spacing of 53±7 nm determined from TEM images. (**c**) Diffraction microscopy image visualizing the scattering upon light reflection from a single stripe in water, where each pixel represents the intensity of light as a function of its propagation direction after reflecting off the stripe as characterized by the polar angle *θ* (measured from the shell surface normal) and the azimuthal angle *ϕ*. The blanked bright spot is an artifact caused by non-avoidable internal reflections in the microscope setup. (**d**) Schematic representation of light reflection from the limpet’s photonic zig-zag multilayer structure for light normally incident on the shell surface. According to Snell’s law, the angle for which the highest reflectivity of a stripe in water is observed, *θ*≈40°, corresponds to an angle of ≈32° (with respect to the incident light) in the calcite shell suggesting an inclination of the reflecting multilayer of *θ’*≈16° with respect to the shell surface, which matches well with the observed characteristic angle of around 144° between the multilayer lamellae. (**e**) Polar plot of reflection intensity showing that each stripe reflects light in a broad angular range of more than a 60° cone angle. This data was obtained by averaging cross-sections of the data shown in **c** at different azimuthal angles *ϕ*. Due to the different orientations of the stripes along the curved shell, portions of the stripe pattern can be clearly seen from almost any direction.

To understand the optical interplay between the multilayer architecture and the underlying colloidal particle array, we investigated several blue stripes using optical microscopy, micro-spectroscopy, and diffraction microscopy. All optical measurements were performed with the shells being immersed and equilibrated in water to emulate the limpet’s natural environment. The reflection from a typical stripe observed from the shell exterior was imaged and spectrally mapped with a resolution of 1 μm (Fig. 4a). The stripe was strongly reflecting and very conspicuous in the wavelength range of 450–570 nm as compared with the surrounding region (Fig. 4a, middle) and was indistinguishable from the neighbouring areas at higher wavelengths (600–800 nm, Fig. 4a, right), as expected due to the low reflectance of the multilayer interference filter in the red and near-infrared spectral range.

Under water, the reflectivity of the blue stripes amounts to 55%±10% (average and s.d., seven limpets, 10–20 measurements on each, Fig. 4b), as compared with a silver reflector (>97% reflectance in the spectral range of interest). The observed spectral distribution matches very well with the theoretical reflectivity calculations that were performed based on a multilayer consisting of calcite lamellae in water with the average thickness, spacing and standard deviation experimentally measured in the limpet’s layered architecture. This observation further supports the hypothesis that the main constituent in the multilayers interstitial space is aqueous. In some cases, a local stripe reflectivity exceeding 75% could be observed.

The stripes scatter blue and green light over a broad angular range. Angle-dependence and wavelength-selectivity of the scattering was further elucidated by imaging a whole stripe’s angular scattering profile using diffraction microscopy with the shell being exposed to normally incident collimated light (Fig. 4c). Each individual pixel in the resulting diffraction image captures the intensity and hue of reflected light that is observed from a specific direction defined by the polar angle *θ* (measured from the sample surface normal) and the azimuthal angle *ϕ*. Three different effects are apparent: (1) the rotational symmetry of the scattering pattern as characterized by the even intensity distribution along *ϕ*; (2) an annulus of high reflection intensity corresponding to *θ*≈40°; (3) the variation of hue with increasing *θ*.

**Figure 5:**
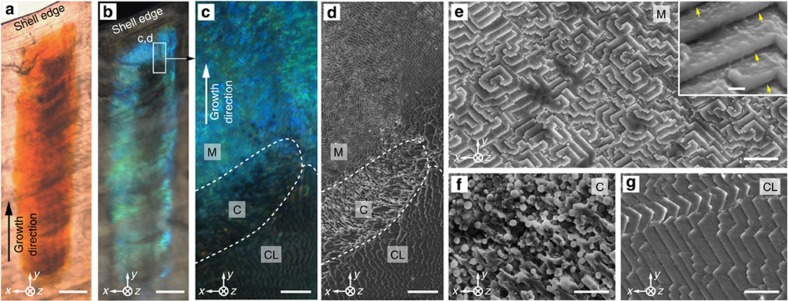
Micromorphology at the growth edge. (**a**,**b**) Transmission and corresponding reflection optical micrographs of a stripe at a shell’s growth edge imaged from the shell interior. Scale bars, 50 μm. (**c**,**d**) High-resolution optical micrograph and SEM image acquired from the transition zone (marked in **b**) between the blue reflecting and non-reflecting areas. Three different regions are apparent: the multilayer region with high blue reflection ‘M’; the region covered with colloidal particles with decreased reflection intensity ‘C’; a cross-lamellar region with very low reflection intensity ‘CL’. Scale bars, 5 μm. (**e**–**g**) High-magnification SEM images of regions of ‘M’, ‘C’ and ‘CL’, respectively. The inset in **e** shows the gaps (yellow arrows) between the lamellae in region M. Scale bars, 2 μm, inset in **e** 200 nm.

The rotational symmetry in the stripes’ diffraction pattern results from local in-plane rotational variations of the multilayer orientation across the stripe, as will be discussed in detail later (Fig. 5e). The annulus of higher intensity at polar angle *θ*≈40° in water suggests that the layered architecture has a predominant inclination of 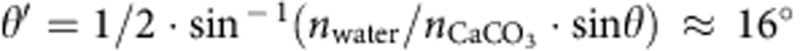 with respect to the shell’s surface normal (Fig. 4d), which correlates very well with the major inclination observed in the zig-zag pattern. Overall, the observed scattering increases the visibility of each individual blue stripe in water, which can be perceived in a range of polar angles of up to >60° measured from the stripes’ surface normal (Fig. 4e; the data in Fig. 4e were obtained by averaging cross-sections of the data visualized in Fig. 4c along different azimuthal angles *ϕ*). Taking into account the macroscopic curvature of the limpet’s shell, at least a portion of the blue stripe pattern can be seen from any direction above the shell (see Supplementary Movie 1).

The change of colour with increasing polar angle *θ* is an effect inherent to the reflection of light from a multilayer where the reflection peak wavelength depends on the incidence angle of light onto the multilayer. For light that is incident normal to the shell surface, reflections that are lying at increasingly higher polar angles (and are increasingly spectrally shifted to lower wavelengths) result from multilayer domains with a higher tilt angle. Consequently, the presence of lower-intensity reflections in the green spectral range that is apparent closer to the centre of Fig. 4c gives evidence for a non-negligible number of multilayer domains that have a smaller tilt angle with respect to the shell surface normal than the majority tilt angle of *θ′*≈16°. The observed colour variation is also evident in a series of spectra obtained by micro-spectroscopy from a single stripe at increasingly larger collection angles using an oil immersion objective (Supplementary Fig. 12).

Knowing that the mineralized multilayer in the stripe region serves as an optical interference filter to produce the strong blue reflection, we will now discuss the role of the disordered array of colloidal particles. Using optical transmission microscopy, we can identify the locations of high abundance of colloidal particles, which correspond to dark areas in the stripe micrographs (Fig. 5a). The same regions also appear dark upon reflection of light from the shell interior (Fig. 5b). A direct correlation of high-resolution optical images (Fig. 5c) and scanning electron micrographs (Fig. 5d) at the interior growth front of the stripe close to the shell edge reveals different morphological regions with distinct optical signatures: At the end of the stripe, closest to the shell’s growth edge, the emergence of the multilayer lamellae is observed (‘M’ in Fig. 5c–e) and the gap spacing among adjacent lamellae can be clearly seen (Fig. 5e, inset). The rotational variation in the planar orientation of the multilayer stacks (Fig. 5e) is responsible for the rotational symmetry observed in the reflection pattern in Fig. 4c. The apparent discrete variations in the in-plane orientation of the multilayer observed in Fig. 5e could explain the ‘granularity’ of the intensity distribution shown in Fig. 4c. Further away from the growth edge (that is, towards older regions of the shell), the lamellae appear to be covered by exposed colloidal particles (‘C’ in Fig. 5c,d,f) with a corresponding decrease in reflection intensity. Even further away from the growth edge, densely packed cross-lamellar structures cover the colloids (‘CL’ in Fig. 5c,d,g) and a very low reflection intensity is observed.

**Figure 6:**
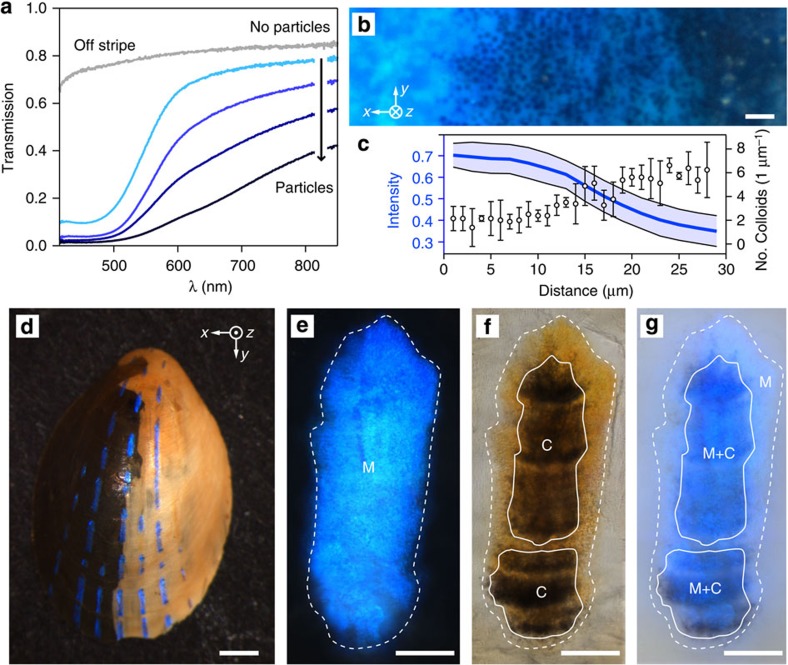
Optical function of the particles beneath the multilayer. (**a**) Light transmission along a path from particle-free zones to particle-covered regions beneath a blue stripe. (**b**) High-resolution optical micrograph acquired in reflection of the transition area from the edge of a blue stripe (no particles, left) towards the centre (dense particle coverage, right). Scale bar, 2 μm. (**c**) Correlation of transmitted light intensity averaged over the wavelength range of 400–750 nm and particle density deduced from SEM cross-sections along a path from left to right of **b**. (**d**) Photograph of a limpet shell with its right interior side painted white. Scale bar, 1 mm. (**e**,**f**) Reflection and transmission optical image of a single stripe before paint application. The circumference of the stripe, that is, the area where the multilayer (M) is located, is marked with a white dashed line. The locations of colloidal particles (C) beneath the multilayer are marked with white solid lines. (**g**) Reflection optical image of the same stripe after painting. The areas with multilayer and colloidal particles (M+C) maintain a strong blue coloration while areas where particles are missing below the multilayer (M) display only a faint blue colour. Scale bars in (**e**–**g**), 50 μm.

The increase of colloidal particle density also leads to reductions in the transmission intensity across the edge of stripes (Fig. 6a). This is further evident by the quantitative negative correlation between the density of colloidal particles (data deduced from FIB cross-sectional images from the stripe edge to its centre) and the transmission intensity (Fig. 6b,c).

While the attenuation of light of complementary colour transmitted through the multilayer in the regions of high colloidal particle abundance could, in principle, result from light scattering by the particles, our experiments reveal that it is primarily due to absorption. This becomes apparent when considering the limpet’s shell together with its underlying soft body. The colour of a live limpet’s body is typically off-white and to imitate this natural colouration during optical characterization, we partially painted the interior of a limpet shell white (Fig. 6d). The blue stripes can be clearly seen in both the unpainted and painted regions without any decrease in intensity or change in hue that would be observed when placing a simple dielectric multilayer mirror on a white background. On a dark background (or with no background at all as is the case in the unpainted part), the reflection of a stripe appears bright blue (Fig. 6e, the region with multilayer (M) is marked by the dashed line). Imaged with transmitted light, the stripe shows up in the complementary colour (orange red), and the dark areas correspond to the locations where the colloidal particles are present (Fig. 6f, areas with colloidal particles underneath the multilayer (C) are marked with solid lines). After the interior of the shell is painted, the blue reflection remains prominent only in regions with particles underneath the multilayer (Fig. 6g, regions marked M+C). In areas deprived of colloidal particles (regions marked M), the light of complementary colour is scattered by the white paint beneath the multilayer and recombines with the blue reflection, resulting in a strong desaturation of the perceived colour. We deduce, therefore, that when present underneath the multilayer, the colloidal particles, which absorb the majority of the transmitted light, provide enhanced contrast for the blue reflection against the white light-scattering soft limpet body below. In addition, the arrangement of the colloidal particles appears to be optimized to ensure complete areal coverage beneath the majority of the multilayer, thus maximizing the absorption of transmitted light (Fig. 2f). The size and separation of the colloidal particles are comparable to other systems that favour multiple scattering^49^, which could increase the number of interactions of the incident light with the absorbing colloidal particles and thereby lead to an increased absorptivity of the thin layer in which the particles are located^50^.

A similar interplay between photonic structure and absorbing and/or strongly scattering particles has been observed in the feathers of birds and the wing scales of butterflies^32^,^51^,^52^. While it has been shown that structural colours can be created by ordered or disordered, appropriately sized and spaced dielectric colloidal particles in combination with a small amount of absorbing material deposited in the particle’s interstitial spaces^53^,^54^,^55^,^56^, the particles in the limpet’s shell do not cause such effects. The size, size distribution and spacing between these particles are too large to generate the observed blue colour. Furthermore, the calcite matrix in which the particles are embedded does not contain appreciable amounts of absorber. In addition, our spectroscopic results demonstrate that the limpet’s blue colour is not purely isotropic in spectral composition and intensity, in contrast to the isotropic structural colours observed in disordered colloidal systems^54^,^55^,^56^. Therefore the absorbing particles underneath the limpet’s multilayer architecture are not the direct origin of the blue colour, which is caused by the multilayer architecture. The particles rather provide an absorbing background for the multilayer filter to enhance the spectral purity of the reflected blue light.

Characterization of the particles’ chemical composition to identify the compounds at the origin of the light absorption remains challenging. While we have some evidence suggesting that the particles might contain melanin (Supplementary Fig. 13), further experiments are required to confirm this observation.

## Discussion

The combination of two mineralized structural elements, that is, the photonic multilayer and the light-absorbing colloidal particles in the underlying calcite matrix, that enable a strong blue reflection with enhanced colour contrast, together with the macroscopic localization of those structures in the form of stripes along the shell, indicate that the photonic system in the blue-rayed limpet might have evolved to serve a purpose in visual communication. Individual stripes on a limpet shell reflect light in a cone opening angle of >120°. Due to the curvature of the shell, each stripe has a distinct orientation on the shell surface and consequently, for most observation positions above the limpet’s shell, there is a set of stripes that can be clearly seen.

**Figure 7:**
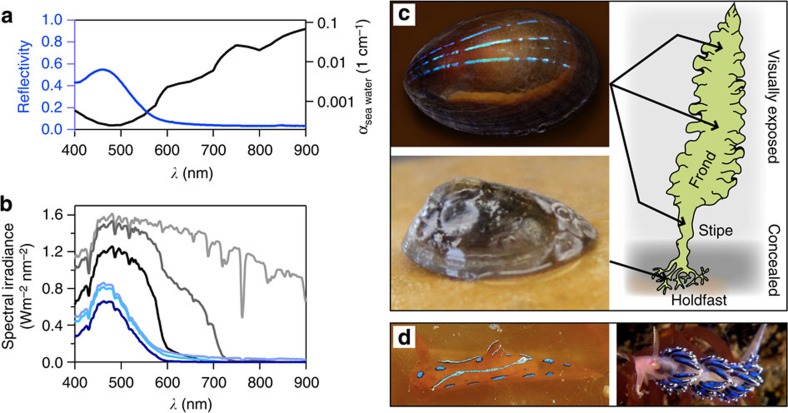
Hypothesis for the functional significance of the blue stripes. (**a**) The stripes’ reflectivity (blue, data set of Fig. 4b) and the linear absorption coefficient of sea water (black, data adapted from ref. 70). (**b**) Spectral irradiance at 0 (ASTM G173-03, 2012), 2 and 10-m depth below sea level before (light grey, grey and black) and after (light blue, blue and dark blue) light is reflected from a blue stripe. (**c**) Comparison of the two morphotypes of *Patella pellucida*. Limpets of the *pellucida* morph (top left) live visually exposed on the kelp fronds, while members of the *laevis* morph (bottom left) are usually found concealed in cavities of the kelp’s holdfast. Top left photograph, acquired with the help of Larry Friesen. (**d**) The toxic and unpalatable nudibranches *Polycera elegans* (left, on kelp) and *Facelina auriculata* (right) with similar blue features and overlapping habitats around the coast of Great Britain and Ireland. Nudibranch photographs courtesy of Josep Lluis Peralta and Jim Anderson.

Besides the wide angular visibility range, the highest intensity of reflection from the stripes is found in the spectral domain that coincides with the absorption minimum of sea water (Fig. 7a). Blue and green light travels farthest in sea water before being attenuated completely and therefore lends itself as the optimum colour range for visual communication in the marine environment (Fig. 7b). At water depths of 10 and 20 m, the intensity of blue light reflected from a limpet’s stripes still amounts to 77% and 60% of its original value at the water surface, respectively, providing good colour fidelity, and therefore allowing the organism to appear highly conspicuous and identifiable even in the deeper depths of its range. In fact, a diver can see the blue colour reflected by limpets on kelp at distances >5 m. This is in stark contrast to other limpet species such as *Helcion pruinosus*^57^ and *Patella granatina*^58^, which display only subtle hints of green iridescence on the exterior of their shells.

It is noteworthy that the limpet *P. pellucida* occurs as two distinct morphotypes. Individuals found on the visually exposed parts of the host kelp, including the fronds and stipes, belong to the *pellucida* morph and display the bright blue stripes over their entire shell (Fig. 7c, top). On the contrary, the *laevis* morph is usually found in concealed cavities of the kelp holdfast, where the environment is darker with low ambient light intensity and is full of visual obstructions. Interestingly, this morph has a stronger, non-transparent shell with pronounced growth lines and lacks the conspicuous blue stripes (Fig. 7c, bottom).

Provided the mineralized photonic structure has indeed evolved for visual communication, what would be the message and to whom would it be addressed? Intraspecific communication seems unlikely. The blue-rayed limpet has very primitive pit eyes just underneath the shell’s apex^59^, which are not appropriately positioned or sufficiently well developed to recognize the blue stripes of conspecifics. From the point of view of interspecific communication advantages, for an herbivorous, slow-moving, largely exposed organism like the blue-rayed limpet that relies on reproduction through broadcast spawning, the largest benefit would lie in preventing predation from other species. This can be achieved by either blending itself into the environment or hiding in concealment as observed for the *laevis* morph. Moreover, the *laevis* morph’s thicker and stronger shell also provides further mechanical protection from potential predators. The thin colourful shell of the highly exposed *pellucida* morph found on the kelp fronds does not blend in at all and is clearly visible, at least to observers with photoreceptors sensitive to the blue spectral range. We speculate that to protect the limpet from potential predators, the stripe patterns and colour might serve to mimic toxic or otherwise distasteful organisms in its habitat that use similar visual features to advertise their unpalatability, a strategy known as Batesian mimicry^60^. Indeed, very similar bright blue features are found on toxic nudibranchs, including *Polycera elegans and Facelina auriculata* (from left to right in Fig. 7d), whose habitats overlap with the limpet’s distribution along the coastal Eastern Atlantic (Supplementary Fig. 14). In addition, from field studies, the *pellucida* morph seems to have developed effective means for keeping the shell surface above and in proximity of the stripes free from fouling so as to maintain the bright blue iridescence, whereas the *laevis* morph usually appears to suffer substantial overgrowth from smaller organisms (compare the images in Fig. 7c). The latter observation further highlights the potential importance of the unique photonic system for the survival of this small and highly conspicuous marine snail.

It is worth noting that the stripes’ reflection shows moderate polarization dependence, which needs to be characterized in more detail. Other marine organisms are known to manipulate the polarization of reflected light for communication, camouflage^61^, and potentially depth gauging^62^. It remains to be investigated whether polarization of reflected light plays any role in the case of the limpet’s blue stripes.

In summary, we show that a composite mineralized structure is responsible for the striking appearance of the blue iridescent stripes on the shell of the limpet *P. pellucida*. Our study clearly identifies various components of the complex photonic system and their role in creating a profound optical effect. We show that the colour arises from the combination of a microscopic mineralized multilayer with regular lamella thickness and gap spacing that creates blue colour by light interference, and an underlying disordered array of absorbing colloidal particles that provide enhanced contrast for the blue hue. The incorporation of this photonic architecture within the continuum of the mineralized shell offers visibility over a broad angular range, high conspicuity due to enhanced colour contrast and the highest spectrally selective reflectivity reported from any marine animal thus far. In contrast to our current study, the diverse range of biological photonic structures investigated previously was found to be predominantly composed of organic materials. It is well known that a number of species demonstrate exquisite control over microstructure, crystallography and macroscopic geometry in the formation of mineralized tissues, thereby constructing materials with impressive mechanical properties. Our discovery of the mineralized hierarchical photonic system localized in the translucent shell of the blue-rayed limpet now shows, for the first time, that a species is capable of taking advantage of complex photonic design principles commonly realized with predominantly organic materials in other species while concurrently exploiting the superior mechanical properties of inorganic materials employed for mechanical protection in the shells of many other mollusk species. This structure likely has evolved to satisfy an optical purpose without overly compromising the shell’s mechanical performance. A growing community of materials scientists and engineers has recently recognized the benefit of biologically inspired and biomimetic approaches towards the design of novel high-performance materials^63^,^64^,^65^,^66^,^67^,^68^. In the context described here, our findings could inspire the design of transparent, mechanically robust, multifunctional optical surfaces with incorporated, controllable display capacity. A more detailed investigation of the morphogenesis of the limpet’s photonic architecture could thus provide new ideas for the technological realization of inorganic photonic materials for a wide range of applications.

## Methods

### Specimens

Limpet specimens were collected along the coastlines of St Abbs (Scotland), the Farnes Islands (North England), Rhosneigr and Fishguard (Wales) in the United Kingdom and were stored under either dry conditions or in 40% ethanol.

### Microscopic studies

For SEM studies using a Helios Nanolab 600 Dual Beam (FEI, OR), the shells were coated with an ultra-thin carbon layer to reduce charging effects before imaging. Cross-sectional samples and TEM samples were prepared using FIB milling with the same system. Final polishing using the ion beam at 2 kV was critical for obtaining a clean surface with a minimum amount of damage. TEM imaging with typical bright-field, dark-field and selected-area electron diffraction techniques was carried out using a JEOL 2011 operated at 120 kV. High-resolution TEM imaging was performed on a JEOL 2010 F operated at 200 kV.

AFM imaging was performed with a Digital Instruments Multimode SPM IIIA (Veeco, CA) (NANOSENSORS Si TMAFM cantilevers, PPP-NCHR-10).

### Synchrotron X-ray nanotomography

X-ray nanotomography was performed at Beamline 32-ID (8.381 keV, APS, Argonne National Laboratory, USA) with samples prepared from FIB cutting (~25 × 25 × 10 μm). The projection slices (pixel resolution, ~13.4 nm) were reconstructed using Xradia TXMReconstructor.

### Optical characterizations

Spectrally resolved intensity maps were acquired for single stripes in a modified Leica DMRX optical microscope with a × 20 objective where an additional photoport allowed for the collection of the light reflected from the specimen into an optical fibre of 50-μm diameter. Spectra were collected from a spot of 2-μm diameter with a collection cone half angle of 33° and the corresponding maps were obtained by laterally scanning the sample in 2-μm steps while maintaining the specimen in constant focus. Quantitative spectral measurements for the determination of the stripes reflectivity in water were performed by using a × 63 water immersion objective with numerical aperture NA=1.0 allowing for the collection of spectra from spots of ~2-μm diameter over a polar angle range of 0–50°. Optical scattering of the samples was studied using a modified Olympus BXFM microscope, where by the incorporation of a Bertrand lens, scattering of a specimen under quasi-plane wave illumination, achieved via an additional photoport, was analysed by imaging the back focal plane of the objective. A high numerical aperture oil immersion objective (Leica PL APO 100 × /1.4 −0.7 OIL) was used to increase the observable angular range. The corresponding scattering angles for water as a medium above the shell surface were then calculated using Snell’s law. These are the angles displayed in Fig. 4c.

### Simulation methods

Theoretical calculations of the blue stripes’ reflectivity were based on an iterative technique for the determination of the reflection coefficients of planar stratified media^69^ implemented in a custom-made MatLab script. To account for the illumination and collection conditions resulting from the use of a water immersion lens with numerical aperture 1.0, for each of the 500 calculation runs, we determined the average reflectivity ‹*R*› of non-polarized light as the weighted average 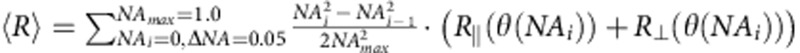, with 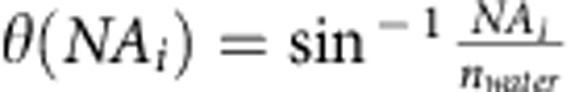 and the reflectivities *R*_||_ of parallel and *R*_⊥_ of perpendicularly polarized light. We also factored in the Gaussian intensity distribution of incident light in the back focal plane of the objective that we mapped before the spectroscopic experiments and calculations.

The reflection curve (shown in blue in Fig. 7a) is the data set re-plotted from Fig. 4b. The absorption curve of water (black curve) was calculated from spectroscopic data reported in the literature (ref. 54 in the paper). In Fig. 7b, the spectral irradiance at sea level (0-m depth) is the ASTM standard (ASTM G173-03, 2012). All other irradiance curves for lower depths were calculated based on this data set taking into account the water absorption (black curve in Fig. 7a). For each depth, the reflected irradiance of the limpet’s blue stripes was calculated by multiplying the corresponding incident irradiance data with the average reflectivity spectrum shown in (Fig. 7a blue curve).

## Author contributions

S.K. and M.K. identified the specimen and conceived the study. M.K. and L.L. performed the optical analysis. L.L., J.C.W., S.K. and M.K. conducted the structural analysis. M.K., C.O. and J.A. oversaw the experiments. L.L., M.K. and J.A. wrote the manuscript. All authors thoroughly revised the manuscript and commented on all aspects.

## Additional information

**How to cite this article:** Li, L. *et al*. A highly conspicuous mineralized composite photonic architecture in the translucent shell of the blue-rayed limpet. *Nat. Commun.* 6:6322 doi: 10.1038/ncomms7322 (2015).

## Supplementary Material

Supplementary Information Supplementary Figures 1-14

Supplementary Movie 1 Limpet shell observation at different angles
