# Supplementary material for: A highly conspicuous mineralized composite photonic architecture in the translucent shell of the blue-rayed limpet
Source: Nat Commun. 2015 Feb 26;6:6322. doi: 10.1038/ncomms7322 (PMC4351589; doi:10.1038/ncomms7322)
Supplement: Supplementary Information — Supplementary Figures 1-14 [file ncomms7322-s1.pdf]

Supplementary Information

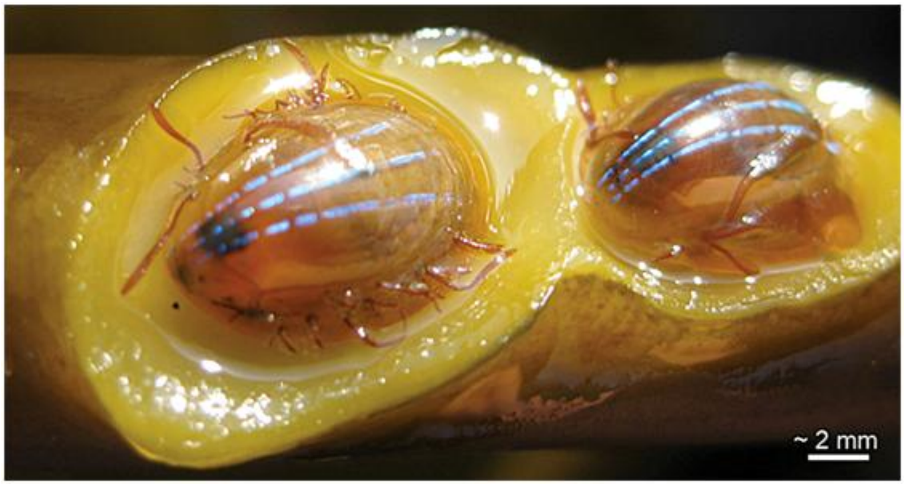

Supplementary Figure 1 | Two blue-rayed limpets in their natural habitat on the stipe of a macroalgae. The surrounding light-yellow regions show exposed algal tissue eaten away by the limpets.

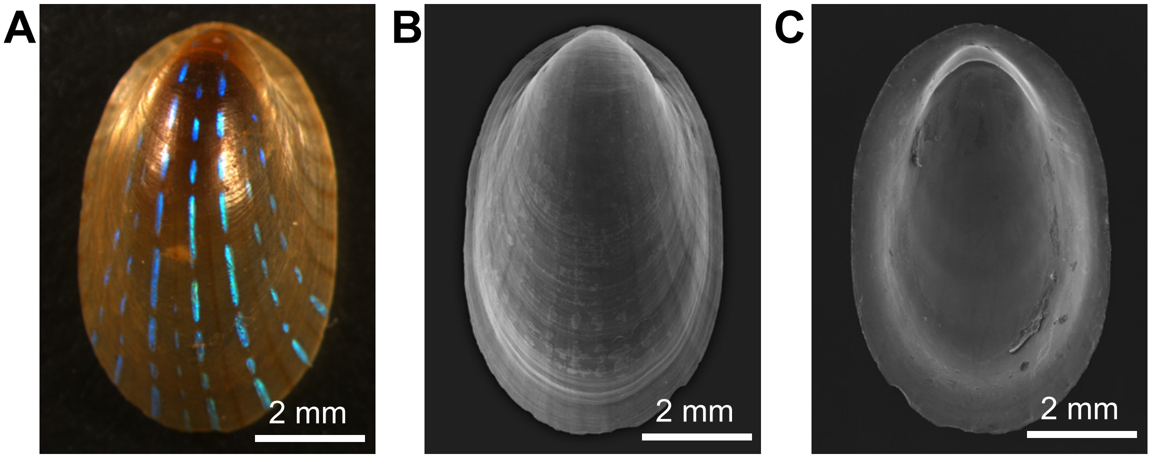

Supplementary Figure 2 | Optical (**A**) and scanning electron micrographs (SEM, **B-C**) showing the reflection of light from the shell exterior (**A**) and the surface topography of the shell's exterior (**B**) and interior (**C**).

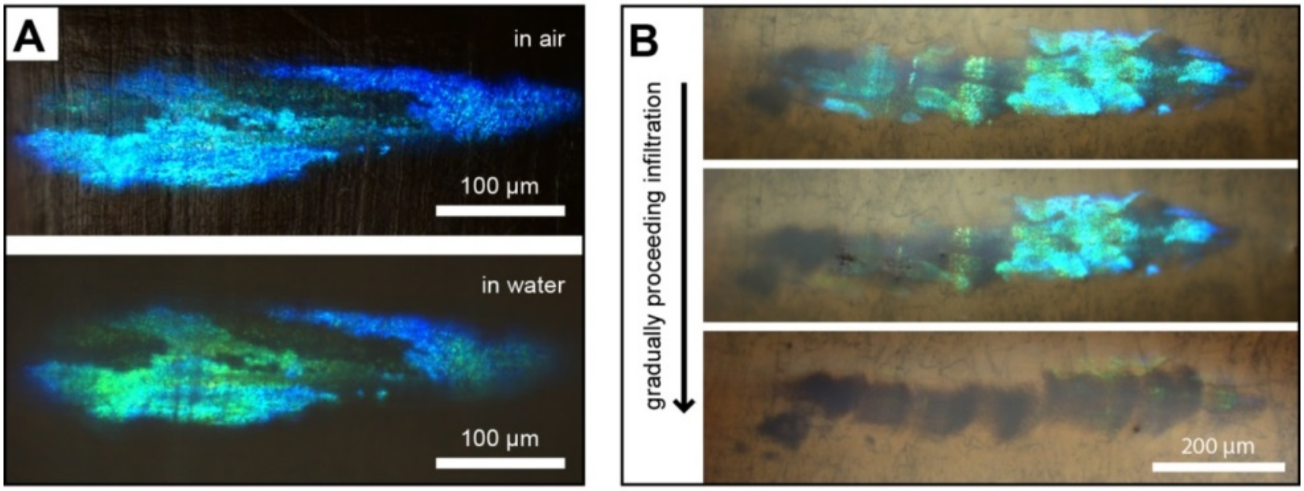

Supplementary Figure 3 | (**A**) A single, partially mechanically damaged stripe shows a pronounced variation of the reflected hue from blue in air towards green when immersed in water. (**B**) Infiltration of index-matching oil into the interstitial spaces of the multilayer in a damaged stripe suppresses the blue reflection and reveals the underlying absorbing particles.

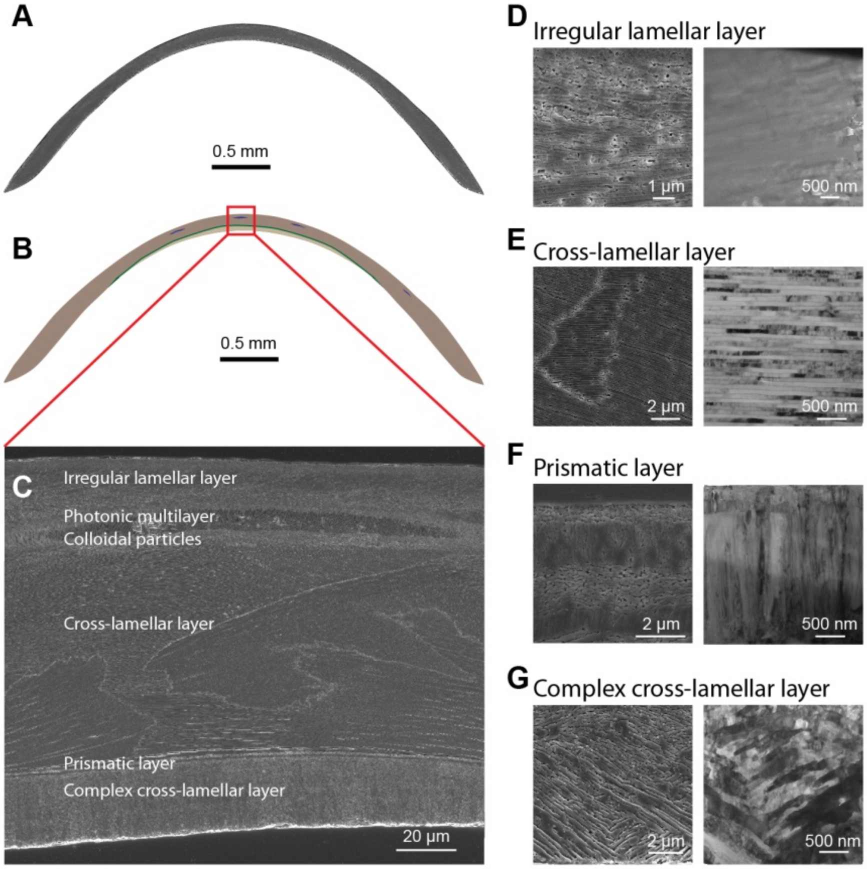

Supplementary Figure 4 | Types of microstructures observed in the shell of blue-rayed limpet. (**A**) SEM image and (**B**) corresponding schematic diagram of the entire shell cross-section. (**C**) Enlarged SEM image of the shell cross-section showing six observed microstructural types: from shell exterior to interior, irregular lamellar layer, photonic multilayer, colloidal particles, cross-lamellar layer, prismatic layer, and complex cross-lamellar layer. Corresponding high magnification SEM and TEM images for the four non-photonic microstructural types are shown in (**D-G**).

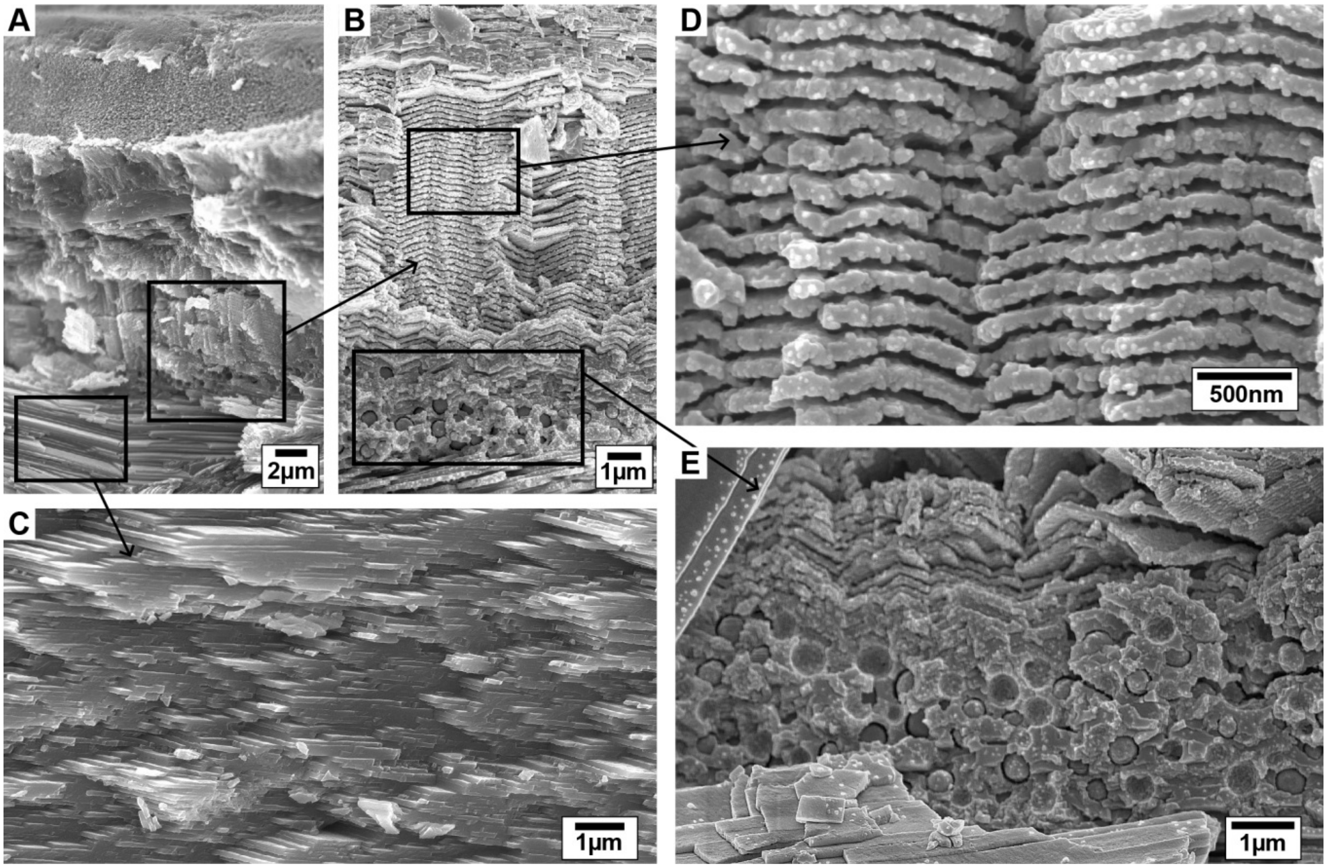

Supplementary Figure 5 | (**A**) Cross-section through the shell of *P. pellucida* obtained by cryo-fracture. (**B**) Section of the photonic multilayer structure and the underlying disordered colloidal particle array. (**C**) Densely packed cross-lamellar calcium carbonate found beneath the colloidal particles. (**D**) Spatially separated calcium carbonate plates with uniform thickness and well-defined spacing found in the areas of the shell with blue stripes. (**E**) Higher magnification view of the disordered colloidal particle array found beneath the photonic multilayer.

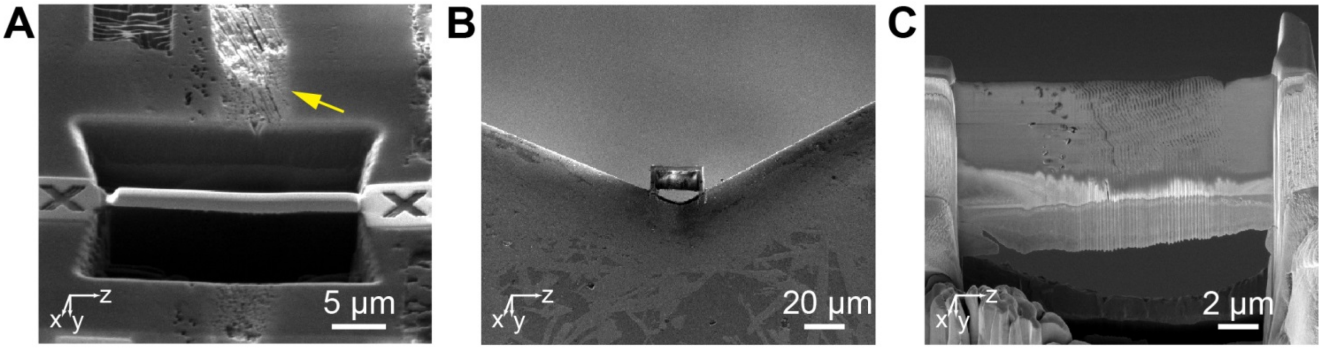

Supplementary Figure 6 | SEM images showing the TEM sample preparation using FIB. (**A**) Two trenches were ion milled, leaving behind a lamella (~ 3 µm thick) running across the multilayer region (arrow). (**B**-**C**) TEM samples mounted on a copper grid by in-situ lifting out using an Omniprobe.

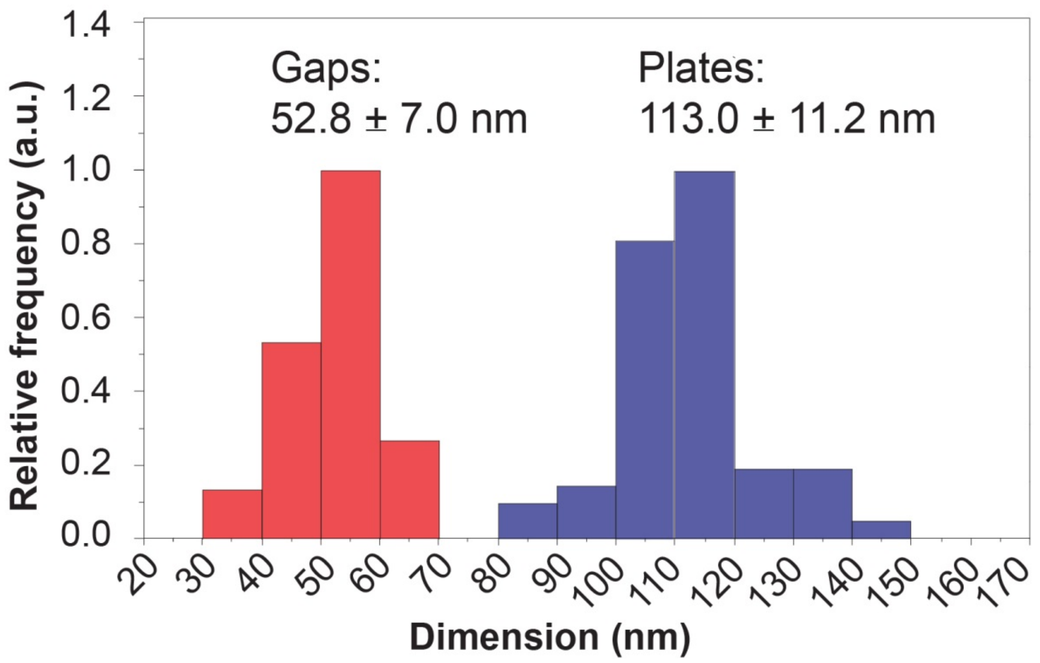

Supplementary Figure 7 | Distribution of calcite layer thickness and gap width within the photonic multilayer.

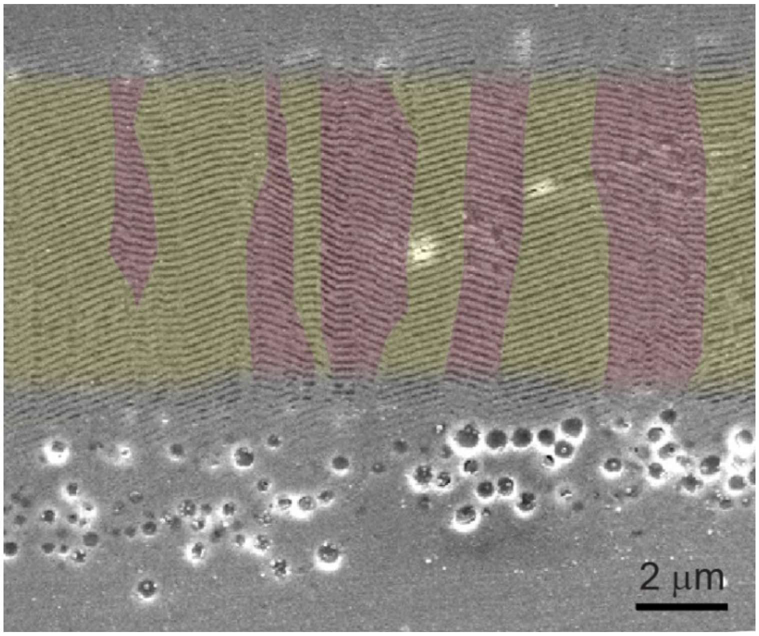

Supplementary Figure 8 | Domains in the zig-zag multilayer architecture. Two different multilayer normal orientations of $\theta' \approx -16^{\circ}$ (pink) and $\theta' \approx 16^{\circ}$ (yellow) with respect to the shell normal surface are visualized in this scanning electron micrograph.

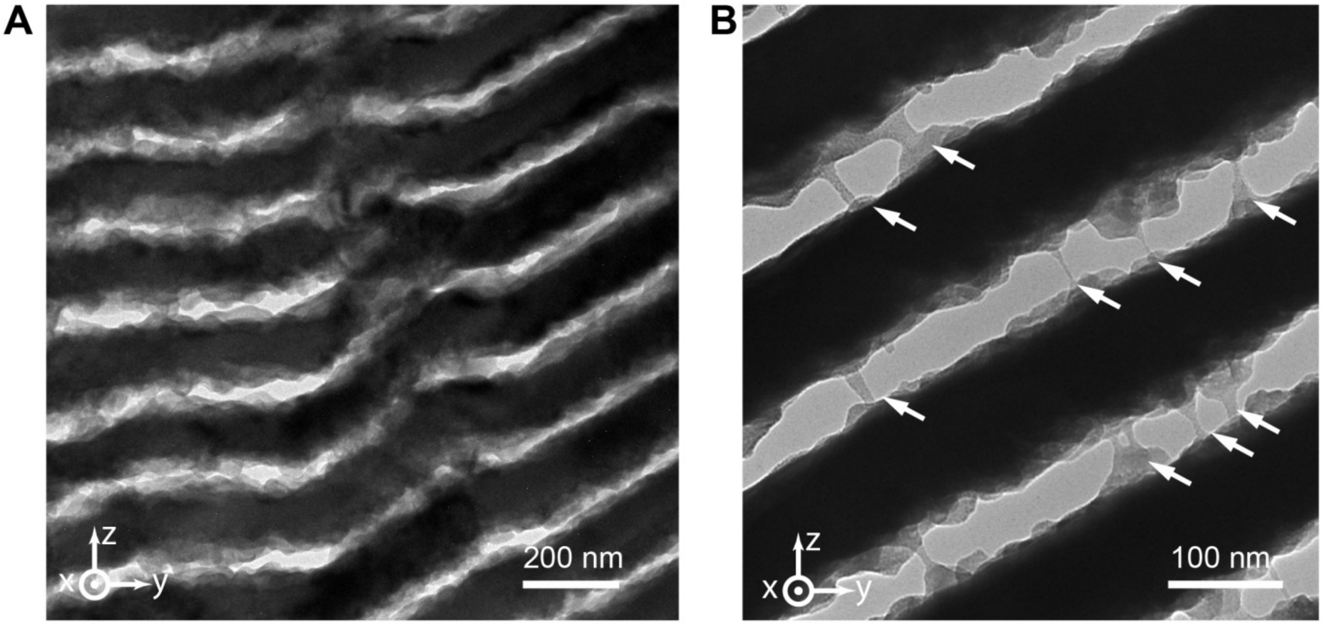

Supplementary Figure 9 |. Structural integrity of the layered architecture is ensured by dislocations (A) and small inter-layer mineral bridges, marked by white arrows (B).

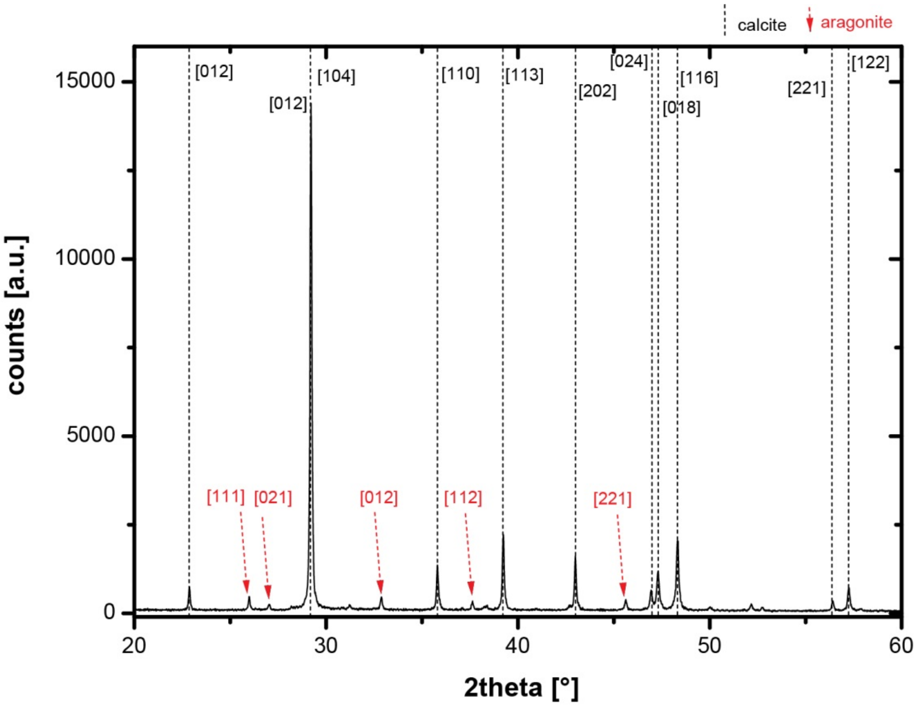

Supplementary Figure 10 | X-ray powder diffraction spectrum of a hand-ground limpet shell, showing the characteristic signatures of calcite and aragonite.

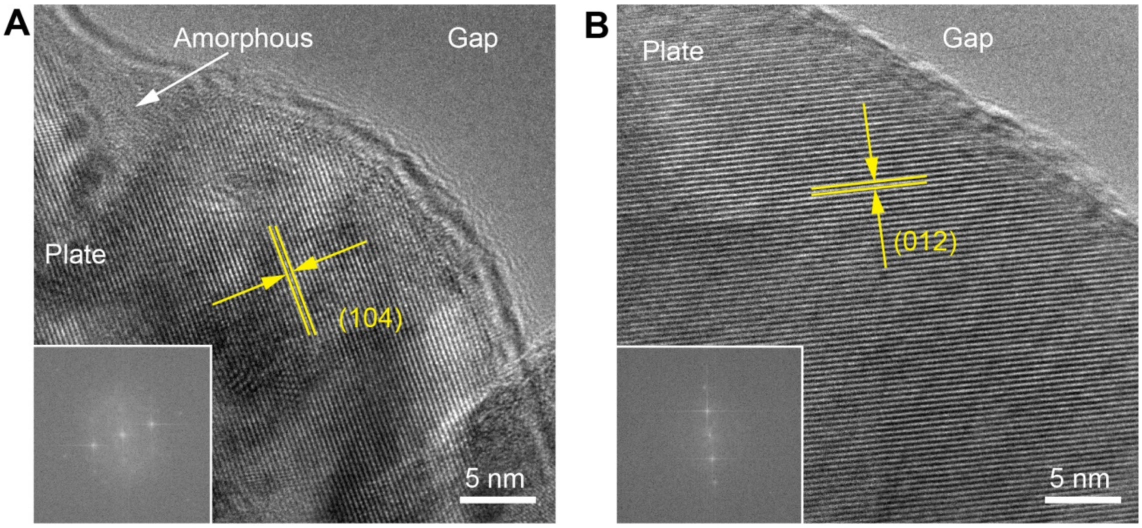

Supplementary Figure 11 | High-resolution TEM images and corresponding FFT patterns (insets) of the crystalline plates in the photonic multilayer structure, where the crystalline regions fully extend to the surface of the plates. A small amorphous region in A is indicated.

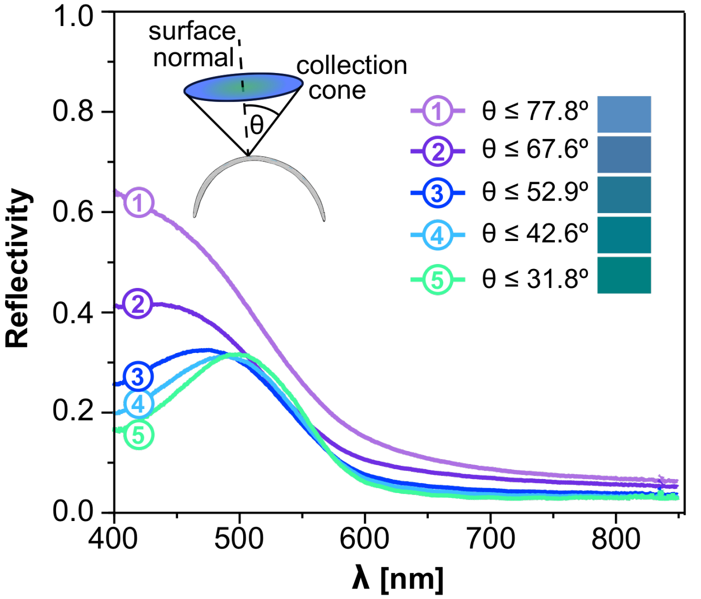

Supplementary Figure 12 | Spectrally resolved reflectivity of a stripe for successively increasing light incidence and collection cone angles, referenced against a >97% reflective silver mirror. Measurements were taken with a Leica 100x oil immersion objective with variable aperture. The angle θ (marked in the inset) signifies the corresponding maximum collection angle for each curve with water assumed to be the exterior medium. The color swatches in the legend show the color resulting from each spectrum. The RGB color values were calculated using the CIE color space algorithm described in (Commission Internationale de L'Eclairage (2004) CIE 015:2004: Colorimetry (Commision Internationale de L'Eclairag, Vienna, Austria)).

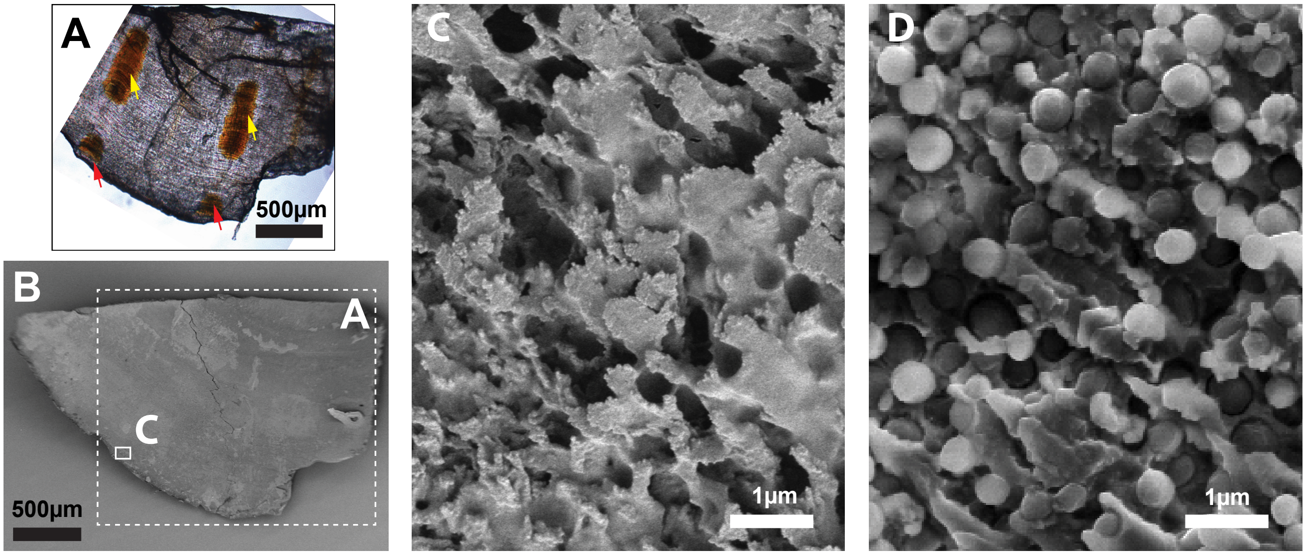

Supplementary Figure 13 | Removal of colloidal particles by bleaching. (**A**) Transmission optical micrograph of a piece of shell after the bleaching experiment. This piece of shell contains both fully developed stripes (yellow arrows) and two developing stripes emerging close to the shell's growth edge (red arrows). (**B**) Scanning electron micrograph of the same piece of shell after bleaching (inner surface). (**C**) High resolution image of the particle area after the bleaching experiment. The particles have disappeared. (**D**) Colloidal particles partially embedded within the calcite matrix before the bleaching experiment.

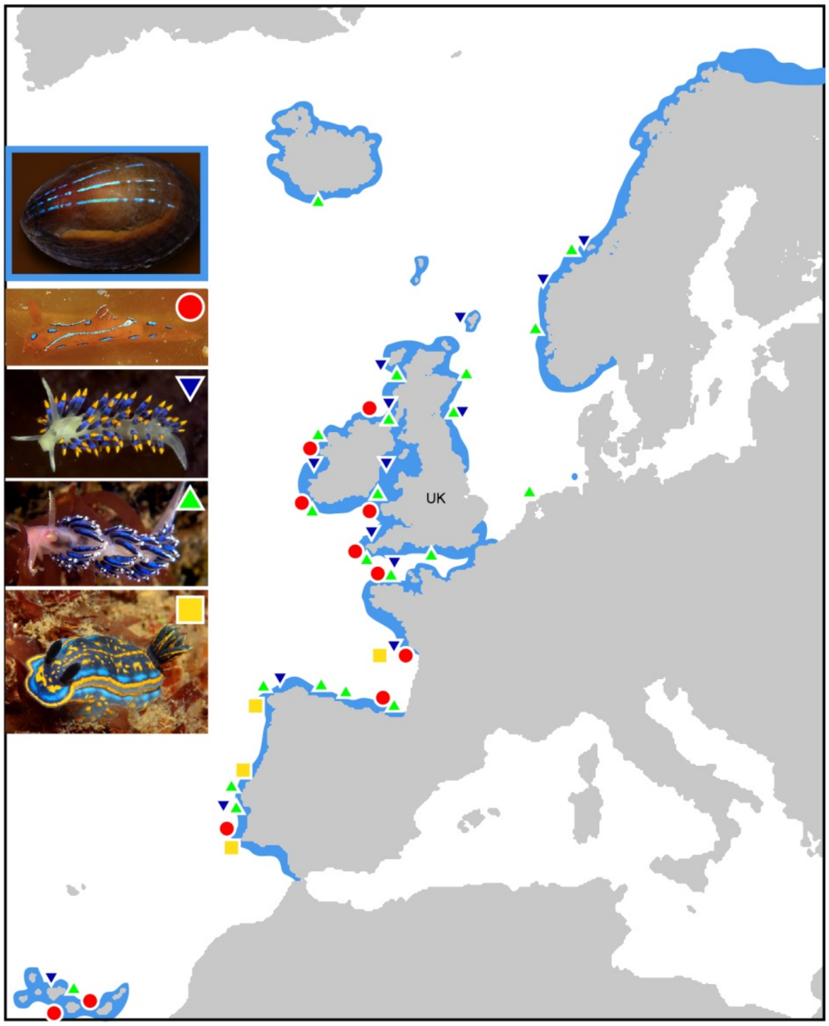

Supplementary Figure 14 | Overlapping geographical distribubutions of the blue rayed limpet, *Patella pellucida* and four toxic nudibranch species (*Polycera elegans* [red circle], *Trinchesia caerulea* [blue triangle], *Facelina auriculata* [green triangle], and *Felimare cantabrica* [yellow square]) which exhibit similar color patterns. Limpet and nudibranch photographs were kindly provided by Larry Friesen, Josep Lluis Peralta, Jim Anderson, and Joao Pedro Silva.
